# Seasonal denning behavior and population dynamics of the late Pleistocene peccary *Platygonus compressus* (Artiodactyla: Tayassuidae) from Bat Cave, Missouri

**DOI:** 10.7717/peerj.7161

**Published:** 2019-07-04

**Authors:** Aaron L. Woodruff, Blaine W. Schubert

**Affiliations:** 1 Florida Museum of Natural History, University of Florida, Gainesville, FL, United States of America; 2 Don Sundquist Center of Excellence in Paleontology and Department of Geosciences, East Tennessee State University, Johnson City, TN, United States of America

**Keywords:** Tayassuidae, Paleoecology, Pleistocene, Demography, *Platygonus compressus*, Denning

## Abstract

The late Pleistocene faunal assemblage from Bat Cave, central Ozarks, Missouri provides an opportunity to assess specific aspects of behavior, ecology, and ontogeny of the Rancholabrean species *Platygonus compressus*. All identifiable elements referable to this taxon were catalogued and examined, and a minimum number of individuals of 73 was determined for the sample. Evidence of seasonal behavioral patterns are reported for the first time in a fossil peccary. Maturation of individuals was assessed using the tooth eruption sequence and occlusal wear patterns for all tooth-bearing mandibular elements and isolated lower dentition. Approximate ages were established through comparison with the extant collared peccary. The presence of distinct, developmentally non-overlapping age groups suggests that *P. compressus* was seasonally present at the Bat Cave locality, with the cave functioning as seasonal shelter in which individuals would occasionally die. The study also suggests the peccaries engaged in synchronous, seasonal breeding behaviors. Demographic assessment of the Bat Cave peccary population suggests that younger individuals formed the bulk of the population at a given time with progressively older individuals becoming scarcer until the age of about 10 years, which matches the typical demographic patterns and life expectancy of extant peccaries.

## Introduction

The large sample of peccary remains recovered from Bat Cave, which have been referred to the late Pleistocene (Rancholabrean) species *Platygonus compressus*, provides the opportunity to examine the paleobiology of this species. Hawksley, Reynolds & Foley (1973) identified 6,339 elements corresponding to a minimum of 98 individual peccaries from Bat Cave, making this the most numerous species recovered from the site. One of us (BWS) visited Bat Cave on several occasions to further assess the deposit. On these trips, additional peccary and other vertebrate remains were collected and a preliminary survey was conducted. This paper focuses on Bat Cave peccary material collected by Hawksley, Reynolds & Foley (1973) and BWS. The Hawksley Collection is curated at the Illinois State Museum, and the material collected by BWS is currently housed at East Tennessee State University. For the present study, the complete Bat Cave Hawksley collection was borrowed from the Illinois State Museum for descriptive and taphonomic analyses.

## Background

### Bat Cave, Missouri

**Figure 1:**
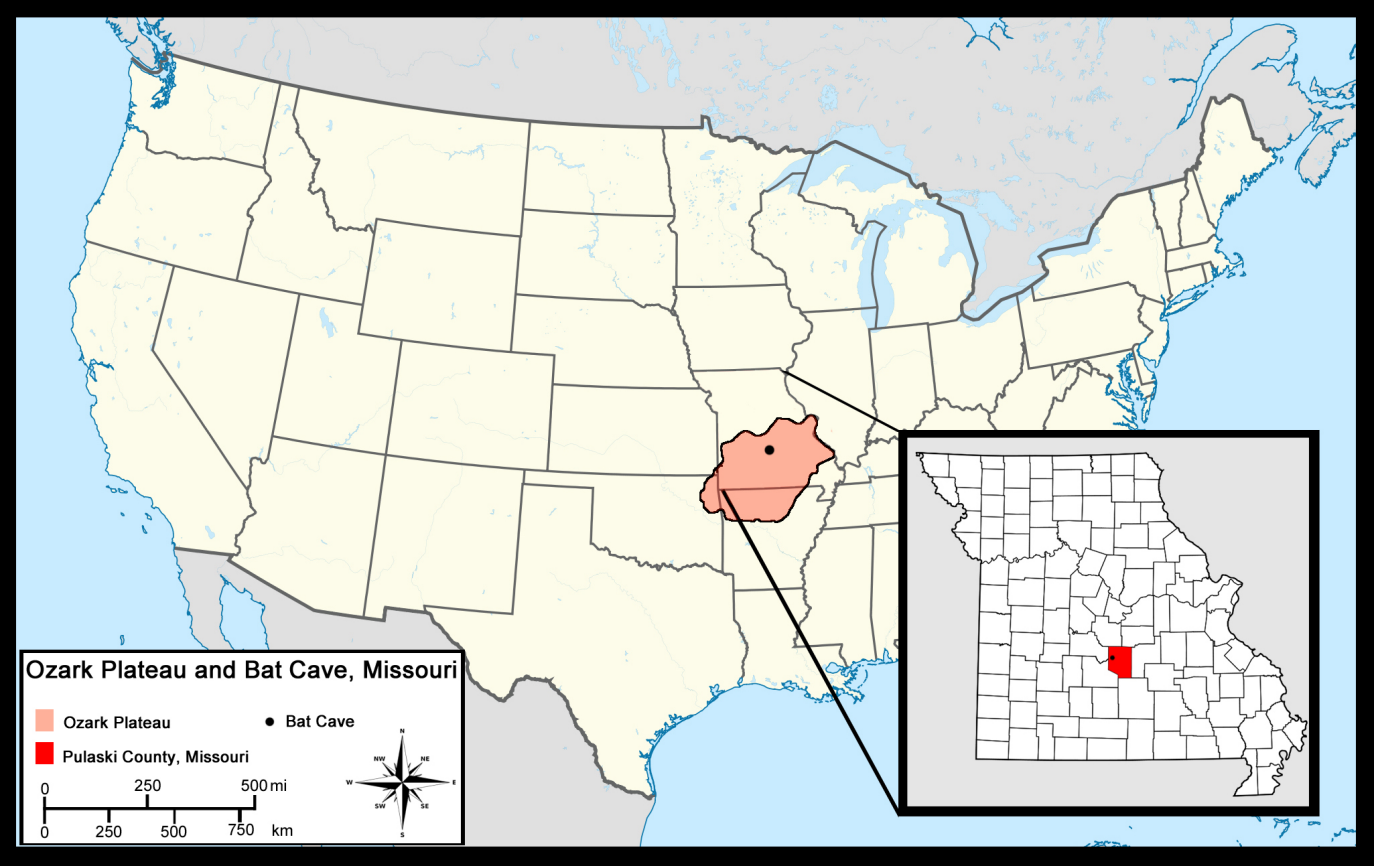
Location of Bat Cave in relation to the Ozark Plateau and Pulaski County, Missouri.

Bat Cave is located 8 km northwest of Waynesville in Pulaski County, Missouri (Hawksley, Reynolds & Foley, 1973) in the central Ozark Plateau (hereafter referred to as Ozarks). It is one of several caves located in Bear Ridge that developed in Ordovician dolomite. The Ozarks are an uplifted region that makes up about half the state of Missouri and portions of northern Arkansas, northeastern Oklahoma, and southwestern Illinois (Fig. 1) (Unklesbay & Vineyard, 1992). Dissolution has led to the formation of numerous karst features such as springs, losing streams, sinkholes, and thousands of caves in the limestones and dolomite bedrock, all of which characterize the region today (Vineyard & Feder, 1974). The Ozarks have remained unglaciated throughout the Quaternary and the region is presently covered in thick, *Quercus*-dominated forests with mixtures of less abundant deciduous trees and a grassland-forest ecotone which has been variable since the Pleistocene (King, 1973; Thom & Wilson, 1980).

Bat Cave itself was recognized for its paleontological potential as early as the late 1950s. Portions of the cave were excavated throughout the 1960s by students and faculty from Central Missouri State University (now University of Central Missouri). Excavations were primarily under the guidance of Dr. Oscar Hawksley and focused on the section of the cave known as the Bone Passage, though fossils were also recovered from Devil’s Kitchen (Hawksley, Reynolds & Foley, 1973, Fig. 2). According to Hawksley, Reynolds & Foley (1973), the Bone Passage is a low, narrow crawlway about 150 m long. The majority of the material was found along the sides of the Bone Passage, though some fossils were found in the center of the floor and consisted of the more durable skeletal elements such as teeth (Hawksley, Reynolds & Foley, 1973). Furthermore, Hawksley, Reynolds & Foley (1973) noted that the terminal end of the Bone Passage (west end) may have been an old entrance (Hawksley, Reynolds & Foley, 1973).

**Figure 2:**
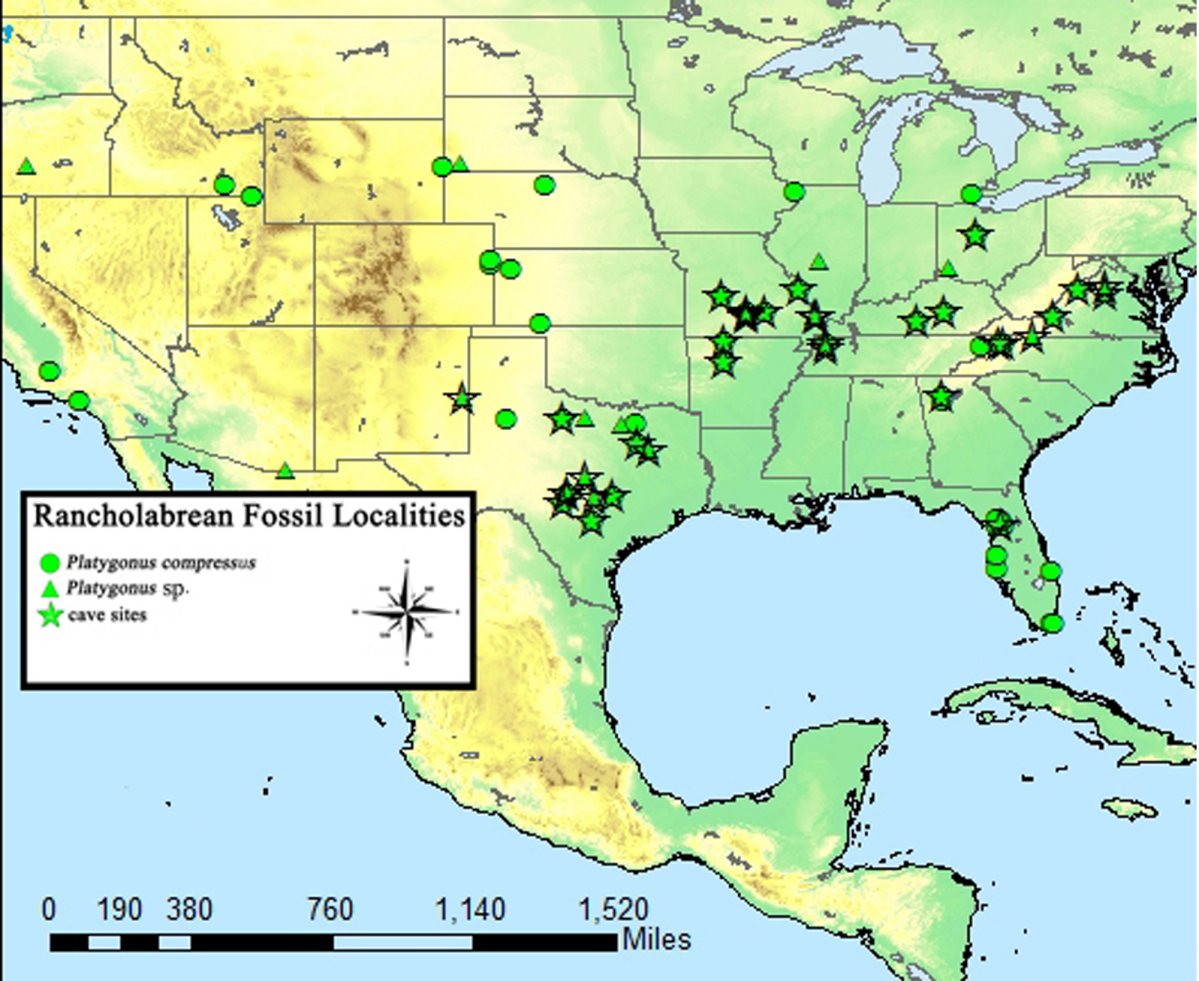
Known Rancholabrean sites which yielded *Platygonus* fossils. Stars denote the occurrence of *Platygonus* in cave deposits. Coordinate data recovered from MIOMAP/FAUNMAP, 2011 (Carrasco et al., 2005).

Hawksley, Reynolds & Foley (1973) suggested the recovered animals likely entered the cave voluntarily or were introduced there by a predator. They also noted it was unlikely that the remains were deposited by water due to the relative lack of fluvial-action abrasion. Following excursions to Bat Cave by BWS, it was noted that a large portion of the deposit is still undisturbed and deserves further attention; specifically, the region closest to the potential Pleistocene entrance and farthest to reach in current passageways.

Hawksley, Reynolds & Foley (1973) identified ∼45 terrestrial and semiaquatic vertebrate taxa, many of which are extant, and based on this interpreted the paleoecology as a well-watered forest-grassland ecotone with a strong taiga influence. Most of the Bat Cave fauna consists of taxa that are extant and are still native to the region. Others, such as the snowshoe hare (*Lepus americanus*), yellow-cheeked vole (*Microtis xanthognathus*), northern bog lemming (*Synaptomys borealis*), and fisher (*Martes pennanti*) are boreal forest taxa whose ranges are far north of the site, suggesting a colder climate at the time of deposition. Extinct taxa include giant short-faced bear (*Arctodus simus*), dire wolf (*Canis dirus*), and flat-headed peccary (*Platygonus compressus*); the latter being not only the most abundantly represented taxon and the subject of the present study, but also the only ungulate reported from the site by Hawksley, Reynolds & Foley (1973).

### Flat-headed peccary (*Platygonus compressus*)

The flat-headed peccary (*Platygonus compressus*) was first discovered in 1806 and described in the mid-19th century (Le Conte, 1848). It was the most common North American peccary species during the late Pleistocene and had a wide distribution, ranging from east to west coast and from Canada to Mexico (Schmidt, 2008; Kurtén & Anderson, 1980). It is particularly abundant in cave deposits around the United States (Schubert & Mead, 2012; Wilson, Guilday & Branstetter, 1975; Hawksley, Reynolds & Foley, 1973; Guilday, Hamilton & McCrady, 1971; Davis, 1969; Slaughter, 1966; Mehl, 1966; Hoare et al., 1964) (Fig. 2), suggesting that such subsurface shelters were important to the ecology of this species.

The large *P. compressus* sample collected from Bat Cave provides the opportunity to obtain demographic and ontogenetic information about the population. Hawksley, Reynolds & Foley (1973) noted that various age groups from very young to very old animals are present in the sample. The three extant peccary species may be defined as territorial and highly social, existing in close knit herds that may hold the same home range over many generations (Taber et al., 1993; Taber, 1991; Bigler, 1974). Through phylogenetic bracketing (Witmer, 1995) it can be inferred that similar behavior was present in *P. compressus*. Further evidence to this end comes in the form of the sheer abundance of *P. compressus* fossils at a given locality, often vastly outnumbering all other taxa (Kurtén & Anderson, 1980; Mehl, 1966), as well as a number of caves with extensive peccary trackways (Schubert & Mead, 2012).

The original minimum number of individuals for the Bat Cave *P. compressus* reported by Hawksley, Reynolds & Foley (1973) was determined to be 98 based on 77 lower left canines and 21 upper left deciduous canines. The MNI was reevaluated in the present study. In addition, skeletal part representation and age demographics were recorded where possible.

## Methods

### Demographic assessment

*MNI*—Canines, the most abundant of all the dental elements, were used to determine the MNI of the BC peccary fauna by Hawksley, Reynolds & Foley (1973). However, the original study failed to elaborate on how canines were differentiated, and some specimens were misidentified. For the present study, comparative morphological observations of the canines were carried out and the MNI was reevaluated and updated based on these criteria. Additional material collected by BWS is also included in this evaluation.

*Sexual Dimorphism*—Sexual dimorphism in peccaries may be determined via certain measurements of the cranium: greatest length, greatest width, and ratio of cranium length to width (Sicuro, Neves & Oliveira, 2011). This method is not possible for the BC population because no intact crania are preserved. Instead, we measured and plotted m3 length against m3 width to test for bimodality. The BC measurements were then plotted together with similar measurements taken for *P. cumberlandensis* from the collections at University of Florida, a taxon that has been demonstrated to possess pronounced sexual dimorphism in its cranial morphology (Wright, 1993). The m3s were selected for study because of their relative abundance and determinate growth. Although bimodality may be observed in peccary canines (Wright, 1993), these teeth were not measured due to their semi-hypselodont nature, irregular placement of the enamel-dentine junction for isolated specimens, and age-related variability in basal width.

*Age & Ontogeny*—Age demographics for the BC peccary population were investigated first by referencing Kirkpatrick & Sowls (1962), in which the age-at-tooth eruption was recorded for extant *Pecari tajacu* (Table 1). We followed this method but focused on the lower dentition only for our age groups. It is assumed that the age-at-eruption does not vary significantly between the extinct and extant taxa. For adult specimens with fully-erupted dentition, tooth wear was also examined as a proxy for age. Special focus was placed on the m3 because it is the last of the permanent dentition to erupt (Kirkpatrick & Sowls, 1962), and tends to be the most hypsodont tooth in the mouths of ungulates (Ozaki et al., 2009); making it a reliable indicator of physical maturity. Similar aging methods have been performed with extant white-tailed deer (Severinghaus, 1949); a taxon whose maximum life expectancy in the wild is comparable to that of extant peccaries. In addition, *O. virginianus* is a reasonable proxy for *P. compressus* because the two taxa are similar in terms of size and presumed browsing or mixed-feeding dietary regime (Schmidt, 2008; Kurtén & Anderson, 1980). Furthermore, the postcanine dental formula in cervids is the same as that of peccaries and the cheek teeth erupt in the same sequence and at a similar rate (Severinghaus, 1949; Kirkpatrick & Sowls, 1962), making these ungulates reasonable analogues for comparison with the BC peccaries.

**Table 1:** This table shows the age at which the lower dentition begin to erupt in the extant collared peccary, *Pecari tajacu*, depicted in days, weeks, and years. Adapted from Kirkpatrick & Sowls (1962).

Tooth	Age (days)	Age (weeks)	Age (years)
dI1	28–49	4–7	0.07–0.13
dI2	45.5–91	6.5–13	0.12–0.25
dC1	At birth	At birth	At birth
dP2	21–35	3–5	0.05–0.1
dP3	14–21	2–3	0.03–0.06
dP4	28–52	4–7.5	0.07–0.14
I1	280–420	40–60	0.7–1.2
I2	364–504	52–72	1–1.4
C1	203–280	29–40	0.5–0.8
P2	462–581	66–83	1.2–1.6
P3			
P4			
M1	119–161	17–23	0.3–0.4
M2	252–350	36–50	0.7–0.96
M3	518–658	74–94	1.4–1.8

## Results

*MN*—Isolated permanent canines can be differentiated by several means. The enamel is generally higher on the lingual side than it is on the labial side, reflecting the slightly angled position of these teeth. In the initial unworn state, the C1 is elliptical in cross-section. As the C1 continues to lengthen and begins to occlude with the c1, a large and flat wear facet develops on the anterior surface of the crown with a distinctive series of parallel grooves running diagonally and downward (ventrally) toward the lingual edge. Left and right C1 can therefore be differentiated by the direction in which these grooves are angled; a right-to-left downward angle indicates a right C1 and a left-to right downward angle indicates the left C1. A secondary facet may also be present on the proximal anterolingual surface. In the initial unworn state, the c1 are triangular in cross-section. The C1 crown and roots are straighter, whereas c1 is more strongly curved its entire length. As it lengthens and begins to occlude with the C1 a large flat wear facet develops on the posterior surface of the crown. Similar to the C1, a series of parallel grooves run diagonally and upward (dorsally) toward the lingual edge of the crown which can assist in identifying the tooth; a right-to-left upward angle indicates a right c1 and a left-to-right upward angle indicates a left c1. A lateral groove may also be present running down the lingual side of the root.

**Figure 3:**
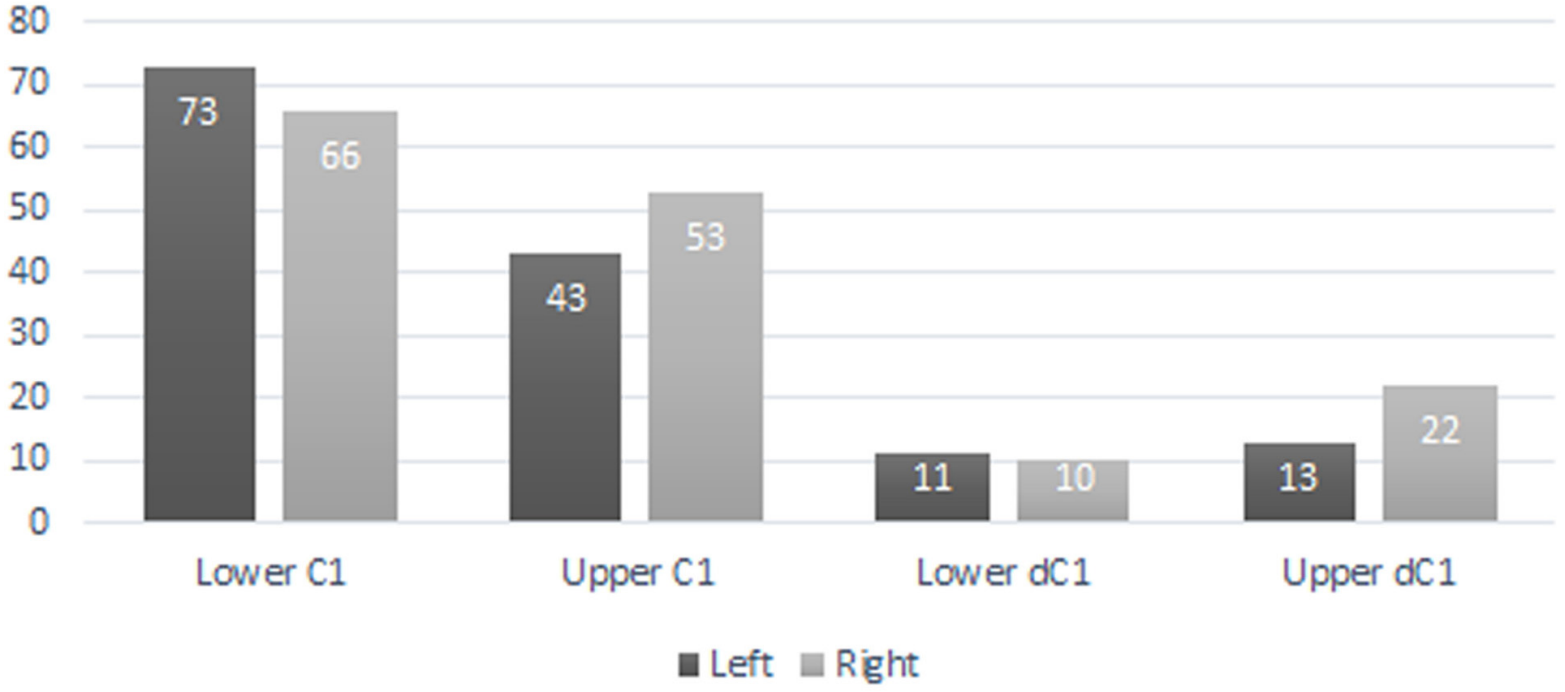
Bat Cave peccary MNI assessment based on the number of isolated and emplaced adult and deciduous canines.

**Figure 4:**
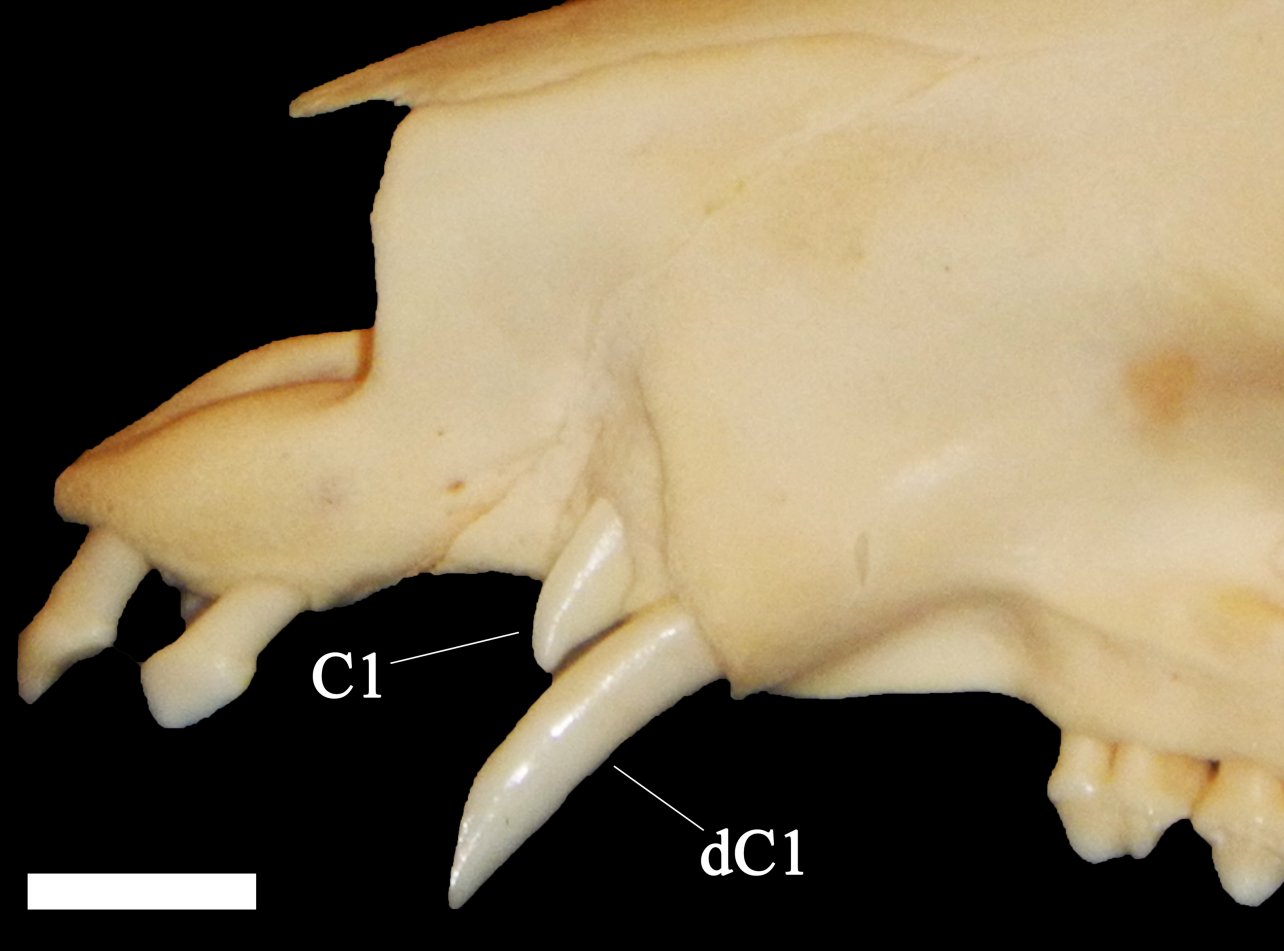
Close-up of a juvenile collared peccary (*Pecari tajacu*) skull ETVP 377 possessing both deciduous canines and the erupting permanent canines. Scale bar = 1 cm.

A total of 207 isolated permanent canines are documented; 59 lower left, 55 lower right, 41 upper left, and 52 upper right. The inclusion of the emplaced canines produces a total of 235 elements; 73 lower left, 66 lower right, 43 upper left, and 53 upper right. A total of 56 isolated deciduous canines are also documented; 11 lower left, 10 lower right, 13 upper left, and 22 upper right (Fig. 3). The present study demonstrated that lower left canines remain the most numerous dental element despite having decreased from the estimation achieved by Hawksley, Reynolds & Foley (1973). The number of upper right deciduous canines reported in the original publication is determined to have been correct. Therefore, based on the methodology performed by Hawksley, Reynolds & Foley (1973), a new MNI of 95 individuals is obtained based on 73 lower left canines and 22 upper right deciduous canines. It must be noted, however, that a considerable number of peccaries from BC are juveniles between the ages of 9 to 12 months old as indicated by tooth eruption sequences and skeletal fusion. At this age, peccaries may possess both permanent and deciduous canines simultaneously, although the latter elements will be in the process of being lost (Fig. 4). This fact raises a problem with the inclusion of both sets of canines in the MNI calculation, as it is possible that a single animal may have been counted twice. Therefore, a more conservative MNI estimate of 73 individuals based solely on the lower left canines is favored. This estimate may change if additional excavations are performed at the BC site.

*Pathologies*—A noteworthy observation in this demographic assessment is the presence of bone pathologies among the BC peccary sample. Among the more interesting pathologies is a juvenile mandible (ISM 499097.8) which exhibits prominent swelling of the post-symphysis left dentary. This swelling does not appear to be the result of impact-related trauma or bone breakage. The potential cause of this pathology has not yet been confirmed, but it is possibly the result of an infection within the bone. Future CT scans may yield further information. Other pathologies include a left lunar (ISM 499122.4) which is highly deformed, possibly due to severe arthritis; a left pelvis (ISM 499141.17) with rugose, deformed bone surrounding the remaining acetabulum; and a proximal phalanx (ETMNH 20226) that shows signs of healing after some antemortem injury.

**Figure 5:**
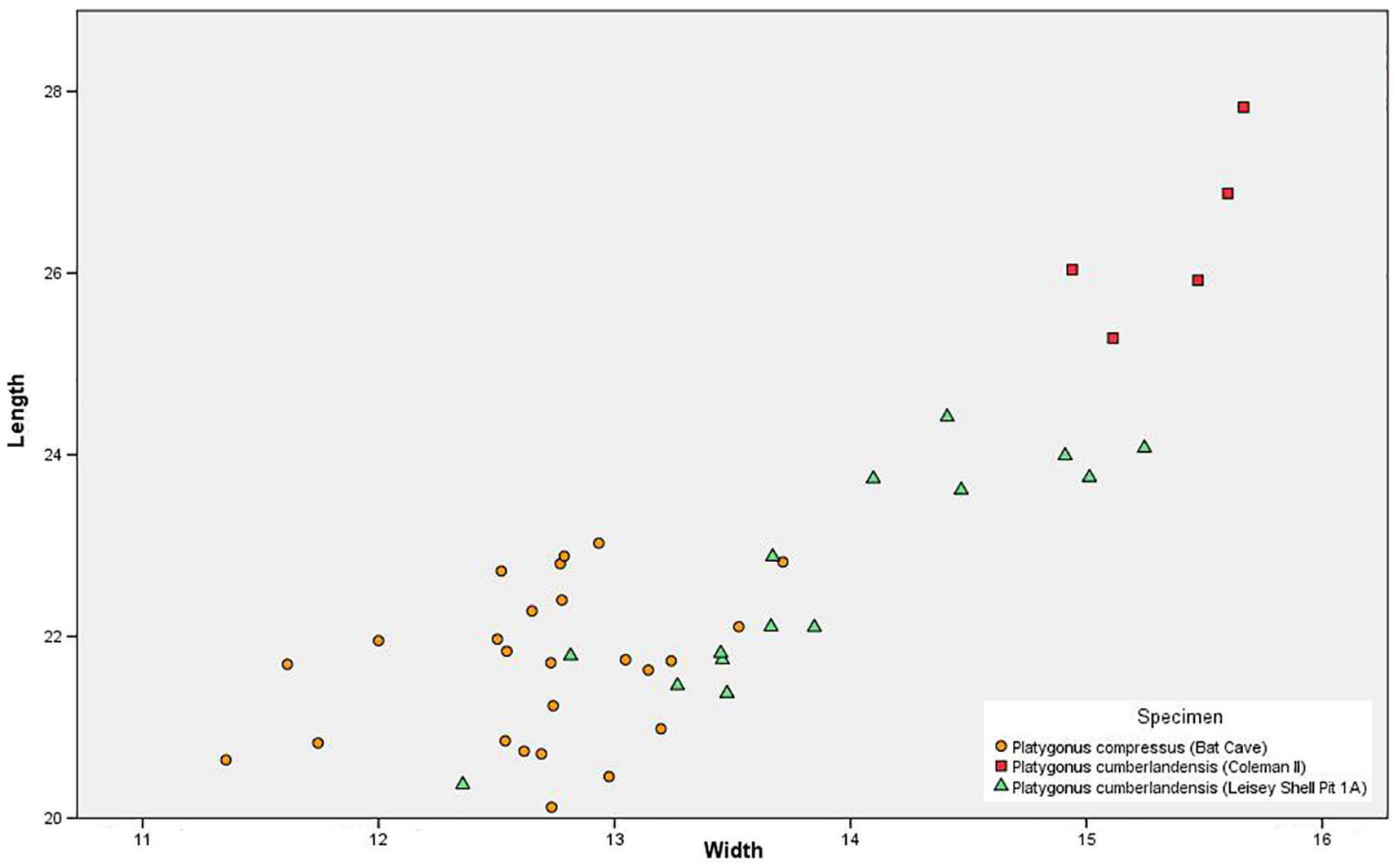
Scatter plot showing m3 measurements of the Bat Cave *Platygonus compressus* (orange circles) as compared to the Irvingtonian species *P. cumberlandensis* from Coleman II, Florida (red squares) and Leisey Shell Pit 1A, Florida (green triangles).

*Sexual Dimorphism*—The m3 measurements failed to identify any notable bimodality among the BC peccaries, thus confirming Wright (1993) conclusion that *P. compressus* displayed minimal sexual dimorphism in terms of size (Fig. 5). Bimodality is noted in *P. cumberlandensis* from Leisey Shell Pit 1A, with larger specimens inferred to be males and smaller specimens inferred to be females (Wright, 1993). The Coleman II *P. cumberlandensis*, however, are shown to plot well above the individuals recorded from Leisey Shell Pit 1A. Such separation between two populations within a narrow geographic range is somewhat unusual and may be attributable to temporal distinction or perhaps the presence of two taxa.

To determine sexual dimorphism among *P. compressus* a different approach may be required. The mandibular keel has been described by Wagner (1903) who believed it to be a sexually dimorphic character, with those of males being particularly “strong and protuberant”. Although this hypothesis is feasible, no comparative analysis has ever been performed to test this. Among the BC sample, 16 mandibles are preserved. One cranium, ISM 499097.2, which we hypothesize to be an older male based on its thickened canines, worn molars, and heavy muscle scarring, bears the most prominent mandibular keel. From the eight juvenile mandibles in the sample, it appears that the keel begins as a rudimentary ridge which increases in prominence with age. A larger collection of intact mandibles is needed to test this hypothesis.

**Figure 6:**
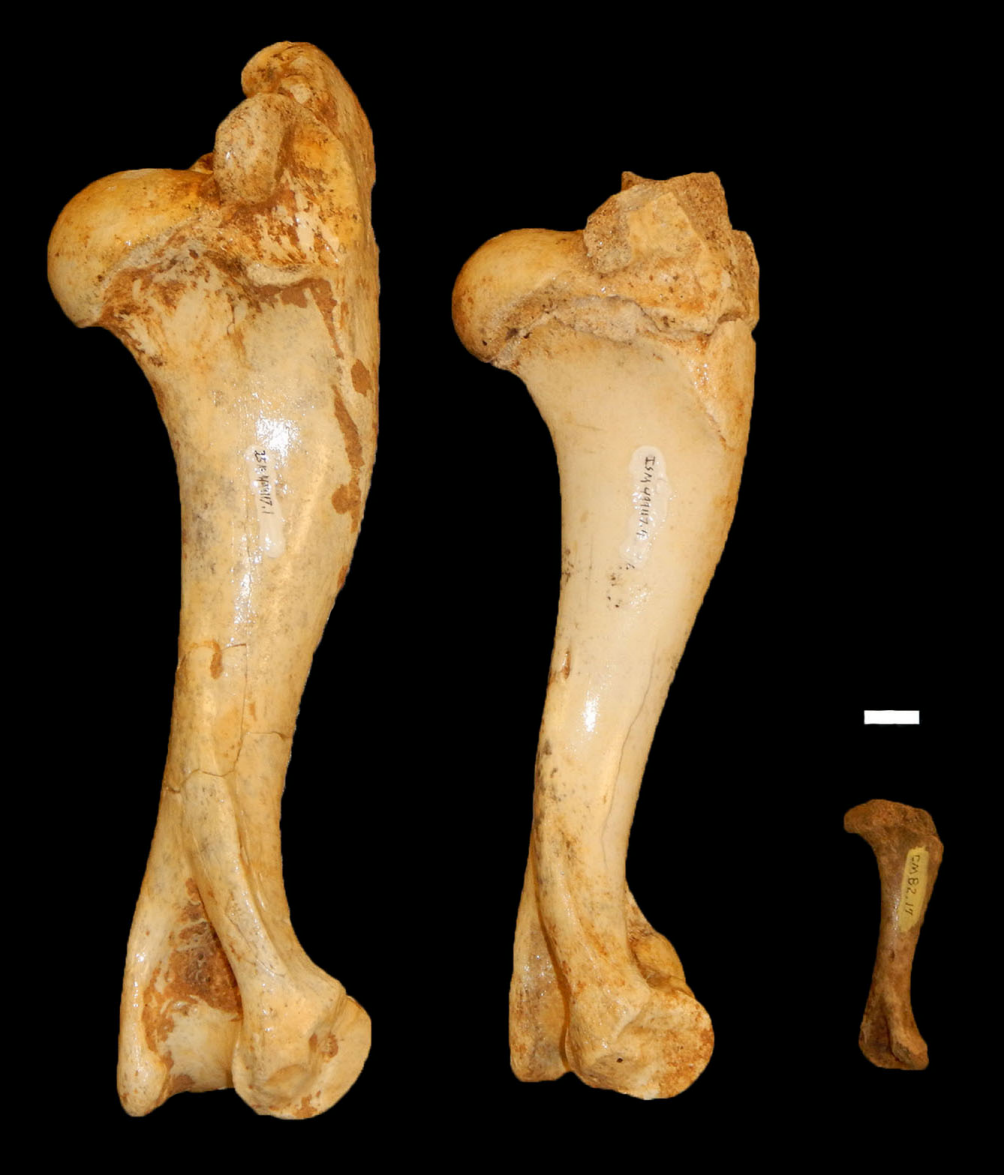
Examples of peccary humeri from Bat Cave which represent three non-overlapping age designations. From left to right: a neonate (ISM 499117.34), yearling (ISM 499117.4), and full grown specimen (ISM 499117.1) of unknown age. Scale bar = 1 cm.

**Figure 7:**
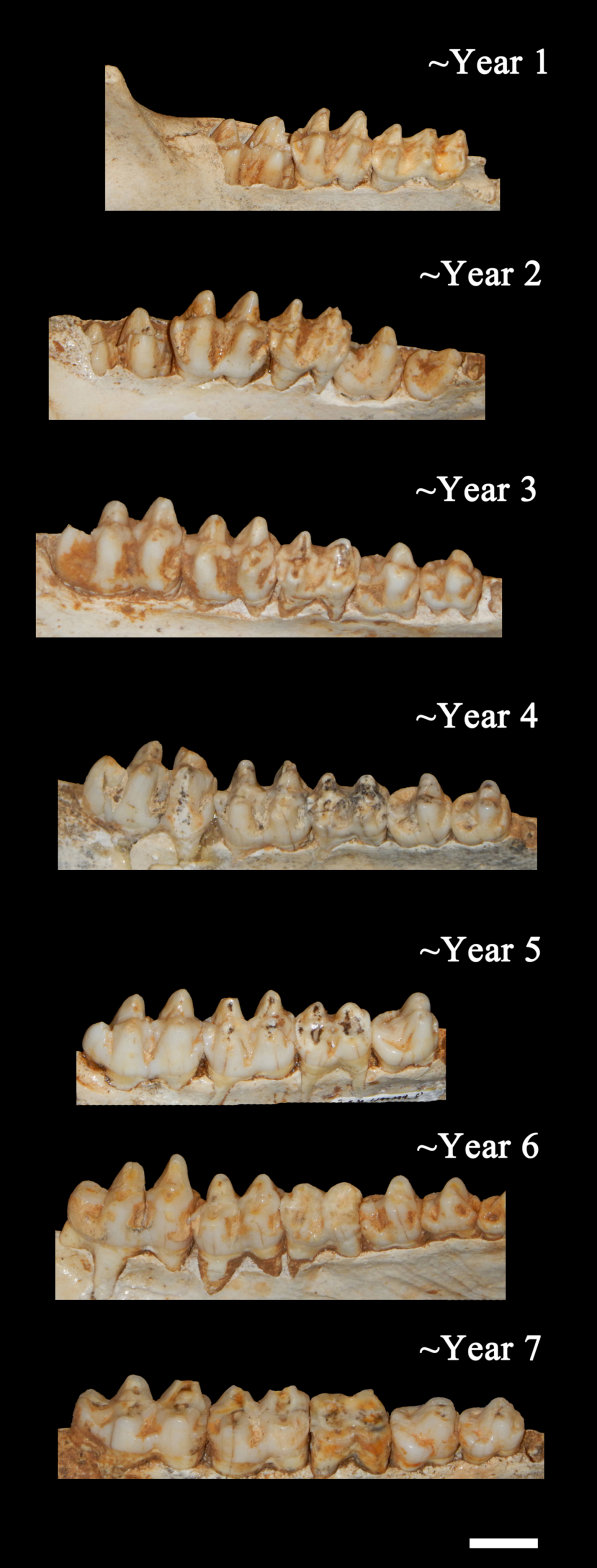
Ontogenetic changes in the lower dentition of the Bat Cave *P. compressus*. Toothrows shown in right-lateral view. Specimens pictured are ISM 499097.15, ISM 499097.6, ISM 499097.4, ISM 499097.1, ISM 499099.10, ISM 499097.7, and ISM 499097.2. Scale bar = 1 cm.

**Figure 8:**
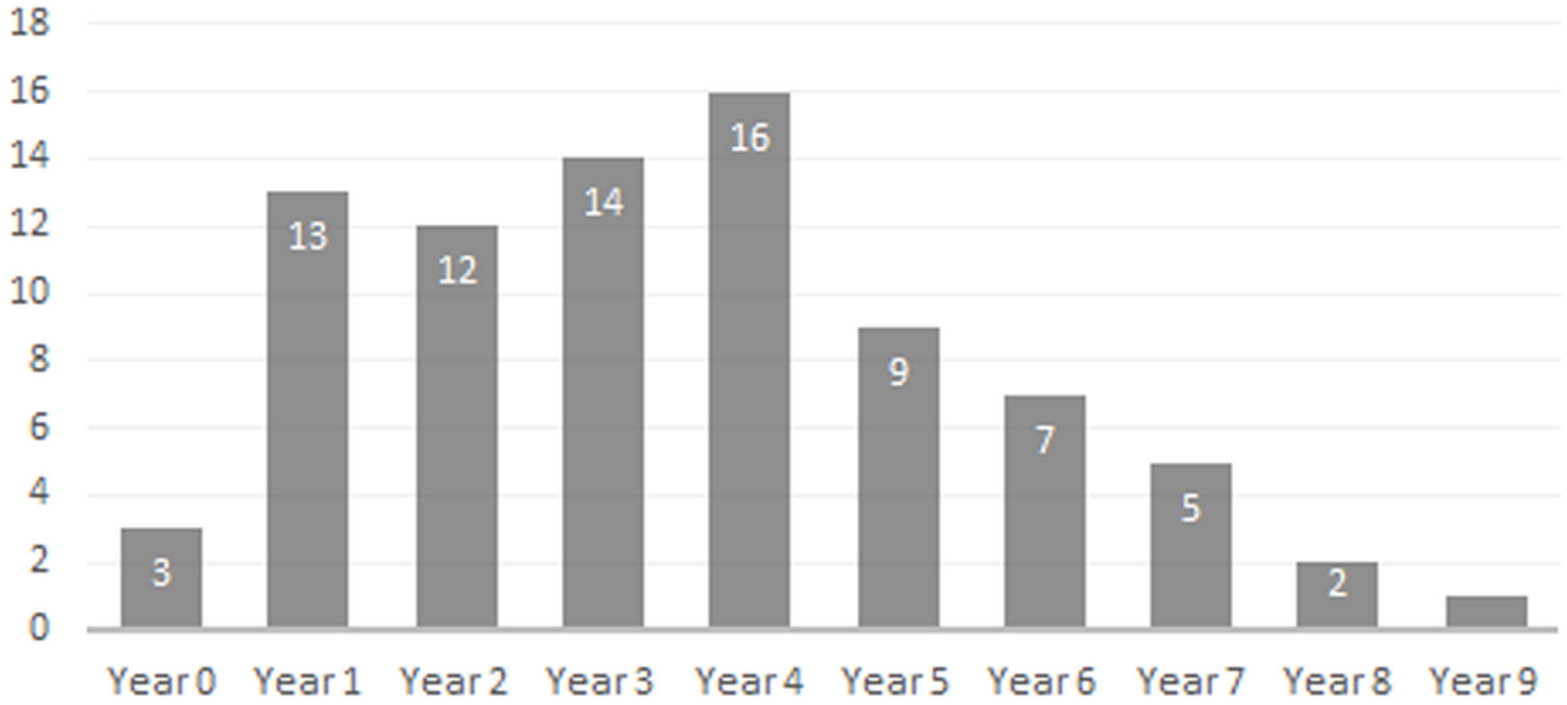
Bat Cave peccary age demographic based on lower dentition.

*Age Demographics*—On initial examination, it is readily evident that every age group from fetuses and newborn infants to elderly adult individuals are represented in the BC sample. On closer examination, the maturation of individuals and age at the time-of-death could be tracked using the eruption sequence as plotted by Kirkpatrick & Sowls (1962) and occlusal wear patterns. Particularly apparent among juvenile or subadult individuals, clearly-definable age groups which show no observable overlap with one another could be established, each group being developmentally separated from each other by approximately nine to 12 months based on Kirkpatrick & Sowls (1962). Similarly, four marked age groups can be observed among the collective limb elements based on size and fusion of the epiphyses (Fig. 6). However, the dentition is recognized as the most useful part of the mammalian anatomy for the purposes of determining age (Kaiser et al., 2009; Skogland, 1988; Spinage, 1973; Morris, 1972; Severinghaus, 1949). Using the lower dentition, and based mostly on complete left or right dentaries, 10 distinct age groupings were established and are defined below (Figs. 7 and 8). Because BC peccary fetuses are only known from postcranial remains, they were not included within the age groupings.

• *Newborn*—Only three specimens are attributable to this age group; ISM 499098.36, ISM 499098.37, and ISM 499099.39. All appear to be within their first month of life, no older than three weeks, as indicated by their extreme small size and erupting deciduous 3rd premolars. ISM 499098.37 appears to be slightly older than ISM 499098.36 due to its larger size and more advanced state of eruption.
• *Year 1*—The c1is partially erupted and is hollow and brittle. Deciduous canine alveoli are usually still present, but the teeth themselves may be absent. Deciduous premolars are present with noticeable wear facets on cusps. The m1 is fully-erupted and exhibits minor wear facets on the labial cusps. In a younger intermediate state, the dc1 would be fully intact and the m1 would be in the process of erupting. The m2 is just beginning to erupt and may not have emerged above the gumline. The masseteric fossa and angular process of the mandible are weakly-developed. Where the posterior portion of the lower jaw remains intact, the m3 bud may be visible within as is the case for ISM 499099.1. Based on these observations, 12 individuals have been placed within this age group.
• *Year 2*—The c1, m1, and m2 are fully erupted and m3 is just beginning to erupt. The dp4 is still present, but has been worn smooth and is weakly rooted in the alveolus as it is gradually being replaced by the underlying p4. Similarly, the remaining deciduous premolars remain present although the permanent premolars may be visible within their crypts where the anterior dentary is damaged, as is the case for ISM 499097.6, ISM 499097.8, ISM 499097.9, ISM 499097.10, ISM 499098.8, and ISM 499099.5. The m1 exhibits noticeable wear facets on the apicies and the m2 cusps retain intact apicies with no evidence of antemortem pitting. The ascending ramus exhibits more prominent muscle depressions and greater depth than that of the previous age group, with some lateral flaring of the ventral edge beginning to take place. Based on these observations, 12 individuals have been placed within this age group.
• *Year 3*—The p2 and m3 have now fully erupted, although the posterior base of the latter may still lie partially in the crypt. The lingual cusps of the m1 have been worn so that they form level transverse ridges with a labial slope, and dentine is now visible unlike what would be observed in a younger individual. The m2 exhibits a similar pattern to that of the year-2 m1 with noticeable wear facets on the apicies, particularly on the labial side. Any wear on the m3 is almost indistinguishable without magnification, particularly the hypoconulid. The c1, when present, shows minimal signs of occlusion with the C1. Further deepening and thickening of the angle has taken place. Based on these observations, 12 individuals have been placed within this age group.
• *Year 4*—The m1 ridges are still distinguishable but have been worn down significantly. The m2 now exhibits wear similar to that of the year-3 m1, forming transverse ridges with a labial slope and visible dentine. The m3 cusps have wear but retain intact apicies with no evidence of antemortem pitting. Based on these observations, 15 individuals have been attributed to this age group.
• *Year 5*—Deterioration of the m1 has progressed so that the anterior and posterior ridges have been worn almost smooth. The anterior and posterior m2 have been worn down further and some pitting may be evident. The m3 apicies are worn considerably but are still distinguishable. Based on these observations, nine individuals have been attributed to this age group with no intermediate tooth wear between it and the previous group.
• *Year 6*—The m1 has now been worn almost completely smooth although there is still some separation between the anterior and posterior cusps. The anterior portion of m1 is worn down to the same level as the posterior edge of the p4 forming a single, continuous surface. The m2 is now in a similar state of wear as the year-5 m1 with its anterior and posterior cusps still forming ridges but bearing pits which expose the dentine. Apicies of the m3 cusps have been worn so that an almost level ridge is formed. The hypoconulid has worn so that a concave depression now exists between it and the posterior cusps. Based on these observations, seven individuals have been attributed to this age group.
• *Year 7*—The m1 is now worn completely smooth with no discernable separation between the anterior and posterior cusps, and the m2 is almost worn smooth with some distinction between the anterior and posterior cusps. The individual cusps of the m3 are still notable but have been worn down with heavy pitting. Based on these observations, five individuals are attributed to this age group.
• *Year 8*—Based on two m3s bearing three distinct, smoothed basins in the former areas of the anterior cusps, posterior cusps, and hypoconulid, the latter now worn to the gumline on its posterior edge. The m1 and m2 are worn smooth. A single m2 which has been worn almost completely smooth may also be attributable to this age group.
• *Year 9*—A single individual has been placed in this group based on a left lower m3 that has become completely worn smooth and dished out with no traces of individual cusps.

## Discussion

*Seasonal Behaviors*—Previous authors have presented evidence of seasonal behavioral patterns in extinct mammal populations based on tooth eruption and wear patterns (e.g., Jefferson & Goldin, 1989). However, the present study marks the first time this principle is applied to fossil peccaries. Extant peccaries occur in close-knit herds which adhere to a set home range and territory over the course of many generations (Taber et al., 1993). Within this area, they will habitually travel to and utilize different locations at regular intervals and at particular times of the year to correspond with the availability of resources (Bigler, 1974). Furthermore, the communal utilization of caves as a means of withstanding temperature extremes is well documented among extant peccaries (e.g., Bissonette, 1978). Phylogenetic bracketing (Witmer, 1995) and mass accumulations of *P. compressus* at numerous fossil localities throughout North America (Schubert & Mead, 2012; Wilson, Guilday & Branstetter, 1975; Hawksley, Reynolds & Foley, 1973; Guilday, Hamilton & McCrady, 1971; Davis, 1969; Slaughter, 1966; Hoare et al., 1964) may indicate these behaviors were present in *P. compressus* as well.

The distinct age groupings reported from the BC peccary sample, each spaced temporally by about nine to 12 months based on tooth eruption (Kirkpatrick & Sowls, 1962), suggests that (1) the cave site was utilized annually and on a seasonal basis, most likely during the winter months when such behavior would be most advantageous and (2) that reproduction for this taxon, at least within the BC locality, was a seasonal occurrence. Aseasonal reproduction is typical among ungulate populations occurring in the tropics and subtropics, which live under constant to near-constant warm temperatures, precipitation, and food availability (Gottdenker & Bodmer, 1998; Rutberg, 1987). Under these conditions, breeding and birthing is often a year-round occurrence with peaks at certain months (Ramos, Pezzuti & Vieira, 2014; Rutberg, 1987). If such a reproductive strategy was utilized among the BC peccary population, we would expect intermediate growth stages between the age groupings identified in the present study.

Seasonal reproduction is a more practical strategy among ungulates occurring in areas of high seasonality, which are subjected to annual fluctuations in temperature, precipitation, and food availability. Cold, wet, and windy weather, combined with food scarcity and the reduced ability of lactating females to produce milk, have been shown to reduce the probability of survival for the newborns of many higher latitude species (Cornell, Hawkins & Hochberg, 1998; Rutberg, 1987; Nowosad, 1975; Slee, 1971). Conception and birthing must therefore be synchronized so that offspring are born during periods where resources are more readily available and conditions are more favorable; thus ensuring that the young-of-the-year are able to gain large body size by autumn to increase the likelihood of winter survivorship (Cornell, Hawkins & Hochberg, 1998; Rutberg, 1987; Clutton-Brock, Guinness & Albon, 1982; Bunnell, 1980; Dauphine & McClure, 1974). This is demonstrated among the BC peccary sample, for which the number of fetal and neonate individuals are extremely rare but the number of yearling specimens is considerably more abundant.

The relative lack of very young juveniles under the age of nine months in the BC peccary sample may suggest that these animals normally did not give birth at the site or, alternatively, that dead offspring were consumed by predators (Munson, 2003) or by adult peccaries (Sowls, 1997) or something else. Following the hypothesis that the local peccaries used the BC site as a winter shelter, it can be inferred that these animals would give birth in early to mid-spring when temperatures were more favorable and vegetation was in abundance. Extant herbivores which live in highly seasonal environments follow the same general reproductive pattern; breeding taking place during early- to mid-fall, gestation throughout the winter, and birthing from mid- to late-spring (Cornell, Hawkins & Hochberg, 1998; Owen-Smith, 1990; Rutberg, 1987; Bunnell, 1980; Dauphine & McClure, 1974; Slee, 1971). The few late-term fetuses and infants identified from the BC peccary sample may then represent early births, or perhaps indicate occasional fluctuations in the annual climate which caused the animals to remain at the site longer than usual.

*Age Structure & Longevity*—The age demographic obtained from the examination of the BC peccary population shows that the composition of animals from one to four years of age was relatively stable, with the number of individuals age five to nine years old gradually declining. This trend matches demographic studies of extant peccaries (Ramos, Pezzuti & Vieira, 2014). As one may expect, relatively young and healthy adult animals form the bulk of the population with older adult individuals steadily dying off due to predation or complications associated with advanced age. In mammals, dental wear is a major factor limiting longevity, with death occurring from the inability to feed effectively and procure adequate nutrients (Kaiser et al., 2009; Ozaki et al., 2009; Skogland, 1988). Along with other ailments associated with old age such as arthritis, progressive tooth wear makes older animals more likely to suffer during periods of food shortage as they are less able to compete with their younger counterparts (Skogland, 1988). For the BC peccary population, this helps explain the downward trend of older adults beyond the age of four. The maximum life expectancy for extant peccaries in the wild is about ten years with most animals dying prior to this due to predation or other natural causes (Cornell, Hawkins & Hochberg, 1998; Owen-Smith, 1990).

## Conclusions

The present study of the BC peccary sample has yielded a revised MNI of 73 individuals based on the number of lower left canines. A larger sample of intact *P. compressus* mandibles is necessary to better assess sexual dimorphism within this taxon. The maturation of individuals was assessed using tooth eruption sequence and occlusal wear patterns for all tooth-bearing mandibular elements and isolated lower dentition, which has demonstrated that all age groups are represented within the sample from unborn fetal to ∼nine-year-old individuals. These age groups are distinctive and non-overlapping, separated developmentally from one another by nine to 12 months. This suggests that *P. compressus* engaged in seasonal breeding behaviors, at least in the BC locality and perhaps other parts of the northern temperate zone. This finding supports the suggestions made by previous authors (Schubert & Mead, 2012; Kurtén & Anderson, 1980) that caves were ecologically important to this species and offers insight into other *P. compressus* cave assemblages. At the time of deposition, the BC site appears to have served as a seasonal, communal shelter for local peccaries, most likely during winter. Demographic assessment of the BC peccary population suggests that subadults and younger adults comprised the bulk of the population and individuals five and older gradually became less abundant. Further taphonomic observations, which will be discussed in a future paper, suggest that dire wolves (*Canis dirus*) hunted or scavenged *P. compressus* inside the cave shelter.

##  Supplemental Information

10.7717/peerj.7161/supp-1 Data S1 DatasetS1 (A) Census of the Bat Cave peccary sample by element. (B) Tally of all isolated and emplaced dentition from the Bat Cave peccary sample. (C) Individual peccary specimens and their approximate age as judged by the state of eruption and wear of the lower dentition. (D) Graph demonstrating the age range of the Bat Cave peccaries based on lower dentition.

## Abbreviations Used

BC: Bat Cave
ETSU: East Tennessee State University
CMS: Central Missouri State University; this institution is now named the University of Central Missouri
ISM: Illinois State Museum
ETMNH: East Tennessee State University Museum of Natural History collections
MNI: minimum number of individuals
NISP: number of identified specimens
C: upper canine
c: lower canine

## Additional Information and Declarations

### Data Availability

The following information was supplied regarding data availability: Census data for the Bat Cave peccary sample is available as a Supplemental File.

## References

[ref-1] Bigler WJ (1974). Seasonal movements and activity patterns of the collared peccary. Journal of Mammalogy.

[ref-2] Bissonette JA (1978). The influence of extremes of temperatures on activity patterns of peccaries. The Southwestern Naturalist.

[ref-3] Bunnell FL (1980). Factors controlling lambing period of Dall’s sheep. Canadian Journal of Zoology.

[ref-4] Carrasco MA, Kraats BP, Davis EB, Barnosky AD (2005). Miocene Mammal Mapping Project (MIOMAP) University of California Museum of Paleontology. http://www.ucmp.berkeley.edu/miomap/.

[ref-5] Clutton-Brock TH, Guinness FE, Albon SD (1982). Red deer: behavior and ecology of two sexes.

[ref-6] Cornell HV, Hawkins BA, Hochberg ME (1998). Towards and empirically-based theory of herbivore demography. Ecological Entomology.

[ref-7] Dauphine TC, McClure RL (1974). Synchronous mating in barren-ground caribou. Journal of Wildlife Management.

[ref-8] Davis LC (1969). The biostratigraphy of Peccary Cave, Newton County, Arkansas. Arkansas Academy of Science Proceedings.

[ref-9] Gottdenker N, Bodmer RE (1998). Reproduction and productivity of white-lipped and collared peccaries in the Peruvian Amazon. Journal of Zoology.

[ref-10] Guilday JE, Hamilton HW, McCrady AD (1971). The Welsh Cave peccaries (*Platygonus*) and associated fauna, Kentucky Pleistocene. Annals of Carnegie Museum.

[ref-11] Hawksley O, Reynolds JF, Foley RL (1973). Pleistocene vertebrate fauna of Bat Cave, Pulaski County, Missouri. Bulletin of the National Speological Society.

[ref-12] Hoare RD, Coash JR, Innis C, Hole TJF (1964). Pleistocene peccary *Platygonus compressus* Leconte from Sandusky County, Ohio. Ohio Journal of Science.

[ref-13] Jefferson GT, Goldin JL (1989). Seasonal migration of Bison antiquus from Rancho la Brea, California. Quaternary Research.

[ref-14] Kaiser TM, Brasch J, Castell JC, Schulz E, Clauss M (2009). Tooth wear in captive wild ruminant species differs from that of free-ranging conspecifics. Mammalian Biology.

[ref-15] King JE (1973). Late Pleistocene palynology and biogeography of the western Missouri Ozarks. Ecological Monographs.

[ref-16] Kirkpatrick RD, Sowls LK (1962). Age determination of the collared peccary by the tooth-replacement pattern. Journal of Wildlife Management.

[ref-17] Kurtén B, Anderson E (1980). Pleistocene mammals of North America.

[ref-18] Le Conte JL (1848). On *Platygonus compressus*: a new fossil pachyderm. Memoirs of the American Academy of Arts and Sciences, New Series.

[ref-19] Mehl MG (1966). Notes on Missouri Pleistocene peccaries. Missouri Speleology.

[ref-20] Morris P (1972). A review of mammalian age determination methods. Mammal Review.

[ref-21] Munson PJ (2003). Age-mediated survivorship pf ungulate mandibles and teeth in canid-ravaged faunal assemblages. Journal of Archaeological Science.

[ref-22] Nowosad RF, Luick JR, Lent PC, Klein DR, White RG (1975). Reindeer survival in the Mackenzie Delta herd, birth to four months.

[ref-23] Owen-Smith N (1990). Demography of a large herbivore, the greater kudu *Tragelaphus strepsiceros*, in relation to rainfall. Journal of Animal Ecology.

[ref-24] Ozaki M, Kaji K, Matsuda N, Ochiai K, Hosoi E, Tado H, Koizumi T, Suwa G, Takatsuki S (2009). The relationship between food habits, molar wear and life expectancy in wild sika deer populations. Journal of Zoology.

[ref-25] Ramos RM, Pezzuti JCB, Vieira EM (2014). Age structure of the Vulnerable white-lipped peccary *Tayassu pecari* in areas under different levels of hunting pressure in the Amazon Forest. Oryx.

[ref-26] Rutberg AT (1987). Adaptive hypothesis of birth synchrony in ruminants: an interspecific test. The American Naturalist.

[ref-27] Schmidt CW (2008). Dental microwear analysis of extinct flat-headed peccary (*Platygonus compressus*) from Southern Indiana. Proceedings of the Indiana Academy of Science.

[ref-28] Schubert BW, Mead JI, White WB, Culer DC (2012). Paleontology of caves. Encyclopedia of caves.

[ref-29] Severinghaus CW (1949). Tooth development and wear as criteria of age in white-tailed deer. The Journal of Wildlife Management.

[ref-30] Sicuro FL, Neves LFM, Oliveira LF (2011). Sex- and age-related morphofunctional differences in skulls of *Tayassu pecari* and *Pecari tajacu* (Artiodactyla: Tayassuidae). Journal of Mammalogy.

[ref-31] Skogland T (1988). Tooth wear by food limitation and its life history consequences in wild reindeer. Oikos.

[ref-32] Slaughter BH (1966). *Platygonus compressus* and associated fauna from the Laubach Cave of Texas. American Midland Naturalist.

[ref-33] Slee J (1971). Physiological factors affecting the energy cost of cold exposures. Proceedings of the Nutrition Society.

[ref-34] Sowls LK (1997). Javelinas and other peccaries: their biology, management, and use.

[ref-35] Spinage CA (1973). A review of the age determination of mammals by means of teeth, with special reference to Africa. African Journal of Ecology.

[ref-36] Taber AB (1991). The status and conservation of the Chacoan peccary in Paraguay. Oryx.

[ref-37] Taber AB, Doncaster CP, Neris NN, Colman FH (1993). Ranging behavior and population dynamics of the Chacoan peccary, *Catagonus wagneri*. Journal of Mammalogy.

[ref-38] Thom RH, Wilson JH (1980). The natural divisions of Missouri: an introduction to the natural history of the state. Transactions of the Missouri Academy of Science.

[ref-39] Unklesbay AG, Vineyard JD (1992). Missouri geology: three billion years of volcanoes, seas, sediments, and erosion.

[ref-40] Vineyard JD, Feder GL (1974). Springs of Missouri. Missouri Department of Natural Resources and U. S. Geological Survey (revised 1982).

[ref-41] Wagner G (1903). Observations on *Platygonus compressus* Leconte. The Journal of Geology.

[ref-42] Wilson RC, Guilday JE, Branstetter JA (1975). Extinct peccary (*Platygonus compressus Leconte*) from a Central Kentucky Cave. The NSS Bulletin.

[ref-43] Witmer LM, Thomason JJ (1995). The extant phylogenetic bracket and the importance of reconstructing soft tissues in fossils. Functional morphology in vertebrate paleontology.

[ref-44] Wright DB (1993). Evolution of sexually dimorphic characters in peccaries (Mammalia, Tayassuidae). Paleontology.

