# Supplementary material for: Seasonal denning behavior and population dynamics of the late Pleistocene peccary Platygonus compressus (Artiodactyla: Tayassuidae) from Bat Cave, Missouri
Source: PeerJ. 2019 Jul 4;7:e7161. doi: 10.7717/peerj.7161 (PMC6612422; doi:10.7717/peerj.7161)
Supplement: Data S1 — (A) Census of the Bat Cave peccary sample by element. (B) Tally of all isolated and emplaced dentition from the Bat Cave peccary sample. (C) Individual peccary specimens and their approximate age as judged by the state of eruption and wear of the lower dentition. (D) Graph demonstrating the age range of the Bat Cave peccaries based on lower dentition. [file peerj-07-7161-s001.docx]

A. Census of the Bat Cave peccary sample by element.

| **Element** | **Left** | **Right** | **Total** |
| --- | --- | --- | --- |
| Mandible (paired dentaries) |  |  | 22 |
| Dentary | 35 | 38 | 73 |
| Palate (paired maxillae) |  |  | 5 |
| Maxilla | 13 | 13 | 26 |
| Frontal | 4 | 4 | 8 |
| Jugal | 8 | 11 | 19 |
| Squamosal | 0 | 0 | 0 |
| Nasal |  |  | 0 |
| Parietal |  |  | 2 |
| Occiput | 0 | 0 | 9 |
| Auditory Bulla | 0 | 0 | 0 |
| Lower dI1-2 | 11 | 9 | 20 |
| Lower dC1 | 11 | 9 | 27 |
| Lower dP2 | 2 | 2 | 3 |
| Lower dP3 | 3 | 3 | 11 |
| Lower dP4 | 6 | 6 | 12 |
| Upper dI1-2 | 7 | 5 | 0 |
| Upper dC1 | 13 | 21 | 26 |
| Upper dP2 | 3 | 1 | 0 |
| Upper dP3 | 2 | 0 | 3 |
| Upper dP4 | 3 | 4 | 7 |
| Lower I1 | 38 | 34 | 72 |
| Lower I2 | 0 | 0 | 0 |
| Lower C1 | 55 | 51 | 106 |
| Lower P2 | 7 | 5 | 12 |
| Lower P3 | 4 | 10 | 14 |
| Lower P4 | 6 | 9 | 15 |
| Lower M1 | 8 | 7 | 15 |
| Lower M2 | 14 | 13 | 27 |
| Lower M3 | 8 | 13 | 21 |
| Upper I1 | 18 | 11 | 29 |
| Upper I2 | 0 | 0 | 0 |
| Upper C1 | 39 | 52 | 91 |
| Upper P2 | 0 | 0 | 0 |
| Upper P3 | 0 | 0 | 0 |
| Upper P4 | 0 | 0 | 0 |
| Upper M1 | 10 | 8 | 18 |
| Upper M2 | 8 | 6 | 14 |
| Upper M3 | 0 | 2 | 2 |
| Scapula | 42 | 50 | 92 |
| Humerus | 38 | 35 | 73 |
| Radius/Ulna | 42 | 41 | 83 |
| Scaphoid | 8 | 8 | 16 |
| Lunar | 4 | 8 | 12 |
| Cuneiform | 7 | 4 | 11 |
| Pisiform | 4 | 1 | 5 |
| Unciform | 3 | 7 | 10 |
| Magnum | 7 | 5 | 12 |
| Trapezoid | 0 | 0 | 0 |
| Trapezium | 0 | 0 | 0 |
| Metacarpal pair | 9 | 7 | 16 |
| Metacarpal III | 15 | 17 | 32 |
| Metacarpal IV | 12 | 16 | 28 |
| Pelvis | 25 | 11 | 36 |
| Femur | 43 | 40 | 83 |
| Tibia | 21 | 28 | 49 |
| Fibula | 18 | 22 | 40 |
| Calcaneus | 31 | 30 | 61 |
| Astragalus | 31 | 21 | 52 |
| Navicular | 9 | 9 | 18 |
| Cuboid | 7 | 10 | 17 |
| Metatarsal pair | 24 | 17 | 41 |
| Metatarsal III | 5 | 6 | 11 |
| Metatarsal IV | 5 | 6 | 11 |
| Patella | 9 | 4 | 13 |
| Prox. Phalanges | 82 | 82 | 164 |
| Med. Phalanges | 53 | 58 | 111 |
| Dist. Phalanges | 48 | 40 | 88 |
| Atlas |  |  | 19 |
| Axis |  |  | 10 |
| CV3-4 |  |  | 29 |
| CV5 |  |  | 7 |
| CV6 |  |  | 11 |
| CV7 |  |  | 11 |
| Thoracic Vert. |  |  | 140 |
| Lumbar Vert. |  |  | 102 |
| Sacrum |  |  | 22 |
| Caudal Vert. |  |  | 25 |
| 1st Sternbra: Manubrium | |  | 8 |
| 2nd Sternebra |  |  | 6 |
| 3rd Sternebra |  |  | 15 |
| 4th Sternebra |  |  | 11 |
| 5th Sternebra |  |  | 4 |
| 6th Sternebra |  |  | 1 |
| Ribs |  |  | 278 |
| Is Fibulae |  |  | 163 |
| Costal Cartilage |  |  | 54 |
| **Total** |  |  | **2693** |

B. Tally of all isolated and emplaced dentition from the Bat Cave peccary sample.

C. Individual peccary specimens and their approximate age as judged by the state of eruption and wear of the lower dentition.

|  | **~Year 0** | **~Year 1** | **~Year 2** | **~Year 3** | **~Year 4** | **~Year 5** | **~Year 6** | **~Year 7** | **~Year 8** | **~Year 9** |
| --- | --- | --- | --- | --- | --- | --- | --- | --- | --- | --- |
| **1** | ISM 499098.36 | ISM 499097.15* | ISM 499097.6 | ISM 499097.4 | ISM 499097.1 | ISM 499099.10 | ISM 499097.7 | ISM 499097.2 | ISM 499229.10 | ISM 499228.7 |
| **2** | ISM 499098.37 | ISM 499097.16* | ISM 499097.9 | ISM 499097.11 | ISM 499097.3 | ISM 499098.9 | ISM 499098.3 | ISM 499228.8 | ISM 499229.11 |  |
| **3** | ISM 499099.38 | ISM 499098.29* | ISM 499097.10 | ISM 499098.5 | ISM 499099.9 | ISM 499099.8 | ISM 499099.6 | ISM 499229.8 |  |  |
| **4** |  | ISM 499097.13 | ISM 499097.5 | ISM 499098.4 | ISM 499099.11 | ISM 499098.10 | ISM 499098.7 | ISM 499229.12 |  |  |
| **5** |  | ISM 499097.12 | ISM 499097.8 | ISM 499099.7 | ISM 499099.19 | ISM 499099.25 | ISM 499099.13 | ISM 499229.9 |  |  |
| **6** |  | ISM 499097.14 | ISM 499098.8 | ISM 499098.6 | ISM 499099.21 | ISM 499229.5 | ISM 499228.6 |  |  |  |
| **7** |  | ISM 499099.4 | ISM 499099.12 | ISM 499099.14 | ISM 499099.20 | ISM 499229.6 | ISM 499228.2 |  |  |  |
| **8** |  | ISM 499099.2 | ISM 499099.5 | ISM 499099.15 | ISM 499099.24 | ISM 499228.5 |  |  |  |  |
| **9** |  | ISM 499099.34 | ISM 499099.27 | ISM 499098.11 | ISM 499099.16 | ISM 499229.7 |  |  |  |  |
| **10** |  | ISM 499099.29 | ISM 499099.26 | ISM 499099.23 | ISM 499098.15 |  |  |  |  |  |
| **11** |  | ISM 499098.21 | ISM 499098.19 | ISM 499229.1 | ISM 499099.22 |  |  |  |  |  |
| **12** |  | ISM 499098.28 | ISM 499229.13 | ISM 499229.4 | ISM 499098.16 |  |  |  |  |  |
| **13** |  | ISM 499098.22 |  | ISM 499228.1 | ISM 499229.3 |  |  |  |  |  |
| **14** |  |  |  | ISM 499228.4 | ISM 499229.14 |  |  |  |  |  |
| **15** |  |  |  |  | ISM 499229.2 |  |  |  |  |  |
| **16** |  |  |  |  | ISM 499228.3 |  |  |  |  |  |

D. Graph demonstrating the age range of the Bat Cave peccaries based on lower dentition.
